# GLP-1 Receptor Agonists in Breast Cancer: A New Frontier in Obesity and Prognosis Management

**DOI:** 10.3390/ijms26167744

**Published:** 2025-08-11

**Authors:** Juliana G. Xande, Auro del Giglio

**Affiliations:** Department of Oncology, Centro Universitário Faculdade de Medicina do ABC (FMABC), Avenida Príncipe de Gales 821, Santo André 09060-650, SP, Brazil

**Keywords:** breast cancer, obesity, GLP-1 receptor agonists, semaglutide, tirzepatide

## Abstract

Obesity is a well-established risk factor for both the incidence and poorer clinical outcomes of Breast Cancer (BC), particularly among hormone receptor-positive postmenopausal women. However, conventional weight loss interventions have yielded limited success in altering cancer prognosis. Recently, glucagon-like peptide-1 receptor agonists (GLP-1 RAs), such as semaglutide and tirzepatide, have emerged as effective pharmacologic agents for sustained weight loss and are under investigation in oncology. This narrative review synthesizes evidence linking obesity to poor BC prognosis and evaluates the therapeutic potential of GLP-1 RAs in this context. Mechanistically, obesity exacerbates tumor progression through hormonal imbalance, chronic inflammation, and adipokine and insulin signaling, targets that may be modifiable through weight reduction. GLP-1 RAs offer multiple benefits, such as appetite suppression, delayed gastric emptying, and enhanced insulin sensitivity. Clinical studies in BC patients have shown weight loss ranging from 2.3% to 5%, likely attenuated by concurrent endocrine therapy. Preliminary data suggest that GLP-1 RA use does not increase the risk of cancer recurrence and may reduce cardiovascular morbidity. However, prospective studies are needed to confirm long-term oncologic safety and efficacy. Disparities in access and cost remain barriers to widespread adoption. Nevertheless, GLP-1 RAs represent a promising adjunct to manage obesity among BC patients, potentially improving metabolic health and long-term cancer outcomes.

## 1. Introduction

Breast Cancer (BC) is the most common malignancy among women and the second most prevalent cancer worldwide. In 2022, an estimated 2.3 million women received a diagnosis of BC, resulting in approximately 670,000 deaths globally [1]. Obesity, however, is even more prevalent. As of 2022, approximately 1 in 8 individuals worldwide lived with obesity [2]. The number of adults with obesity will rise from 524 million in 2010 to about 1.13 billion by 2030, an increase of more than 115% [2].

Obesity is a chronic, multifactorial disease characterized by excessive fat accumulation that poses significant health risks. Assessed using body mass index (BMI), calculated as weight in kilograms divided by height in meters squared, a BMI ≥ 30 kg/m^2^ defines obesity, while a BMI between 25 and 29.9 kg/m^2^ represents overweight [2].

Given the high prevalence of both BC and obesity, their coexistence is unsurprisingly common. For instance, a Swiss study reported that at the time of BC diagnosis, 32.3% of women were overweight and 19.6% were obese; thus, over half of the patients were above a healthy weight [3]. In the United States, from 2011 to 2014, approximately 35% of premenopausal women aged 20 to 59 were obese, and about 20% of BC cases occurred in women with obesity [4]. More recently, a 2023 meta-analysis including 41,049 premenopausal BC patients found that 29% were overweight and 17.8% were obese at diagnosis, reinforcing the high prevalence of excess weight in this population [5]. Moreover, women undergoing active treatment for BC tend to gain an average of 3 kg per year [6].

Obesity has been consistently associated with an increased risk of BC incidence and poorer clinical outcomes, particularly in postmenopausal women with hormone receptor–positive disease. Obesity increases the risk of recurrence and mortality in BC patients by 35–40%, making it a significant negative prognostic factor across subtypes [7]. However, whether obesity represents a modifiable prognostic factor remains unclear, given the limited effectiveness of traditional weight management strategies (e.g., diet and exercise) in producing substantial and sustained weight loss or improving cancer outcomes [8]. Similarly, despite inducing more dramatic weight loss, there is little evidence that bariatric surgery improves oncologic outcomes in BC patients with obesity [9].

The recent development of potent pharmacologic agents capable of achieving significant and sustained weight loss—such as the glucagon-like peptide-1 (GLP-1) receptor agonists semaglutide and tirzepatide—introduced a new research frontier [10,11,12]. These agents, along with others under investigation, could alter the future landscape of obesity management. Their superior weight loss efficacy of these drugs may provide an opportunity to mitigate the adverse prognostic impact of obesity in BC [13]. This review aims to evaluate the existing evidence on the prognostic role of obesity in BC patients and assess current data regarding the efficacy and safety of GLP-1 receptor agonists (GLP-1 RAs) in women with overweight or obesity with BC.

## 2. The Relationship Between Obesity and Breast Cancer Prognosis

### 2.1. Impact of Obesity on Breast Cancer Outcomes

Obesity has a well-documented negative impact on BC outcomes through multiple biological and clinical pathways. It is associated with a 35% to 40% increased risk of BC recurrence and mortality, particularly among patients with estrogen receptor-positive tumors. Conversely, the correlation between obesity and outcomes in triple-negative and HER2-positive subtypes remains less conclusive [7]. A meta-analysis of forty-three studies involving women with early-stage BC found a 33% increase in BC-related and overall mortality among women with obesity compared to their non-obese counterparts [14,15]. A recent Japanese cohort study of 3380 premenopausal women with hormone receptor-positive BC found that those with a BMI ≥ 25 kg/m^2^ (overweight or obese) had significantly worse BC-specific survival compared to women with normal or low BMI. This association remained significant among those treated with tamoxifen alone [16]. Aside from BC, obesity is associated with less favorable outcomes in several types of cancer. Pati et al. reported that excess body fat is associated with an approximately 17% increase in cancer-specific mortality [17].

### 2.2. Weight Gain During Breast Cancer Treatment

Weight gain is common among women undergoing treatment for early-stage BC, particularly those receiving chemotherapy, and it may contribute to worsened prognosis [18]. Estimates suggest that 50% to 96% of patients gain weight during or after treatment, with average increases ranging from 1 to 5 kg within the first year [19]. Approximately 20% may gain over 10% of their body weight [6], further complicating an already challenging clinical and emotional journey.

There are multiple factors that can lead to weight gain associated with BC treatment. Chemotherapy can lead to fatigue and reduced physical activity, early menopause, metabolic slowing, and fat accumulation. Hormonal and metabolic changes—such as insulin resistance, increased adipokine levels (e.g., leptin), and chronic inflammation—further alter energy balance. Corticosteroids, commonly used during treatment, also promote weight gain through appetite stimulation and fluid retention [6].

### 2.3. Mechanisms Linking Obesity to Poor Prognosis

Obesity worsens BC prognosis through interconnected biological mechanisms. These include hormonal dysregulation, chronic inflammation, altered adipokine signaling, insulin resistance, and activation of oncogenic intracellular pathways that collectively promote tumor progression, metastasis, and therapy resistance [4,20].

In postmenopausal women, excess adipose tissue increases aromatase activity, enhancing the conversion of androgens to estrogens, which can stimulate the proliferation and survival of estrogen receptor-positive BC cells [4,20]. Obesity is also associated with a pro-inflammatory state marked by elevated levels of cytokines such as TNF-α, IL-6, and prostaglandin E2, which promote tumor proliferation, invasion, angiogenesis, and immune evasion [4,20].

Moreover, obesity disturbs adipokine balance. Elevated leptin levels activate signaling pathways (JAK/STAT, MAPK, and PI3K/Akt) that support cancer cell proliferation, angiogenesis, and metastasis. In contrast, adiponectin—an adipokine known for its anti-inflammatory and antiproliferative properties—decreases in cases of obesity [4,20].

Insulin resistance and resulting hyperinsulinemia, along with elevated insulin-like growth factors (IGFs), stimulate mitogenic signaling (PI3K/Akt/mTOR and MAPK), promoting tumor growth and survival. These mechanisms contribute to a pro-tumorigenic, therapy-resistant microenvironment [4,20].

Gene expression profiles in tumors from patients with obesity often show upregulation of inflammatory genes and downregulation of those involved in drug metabolism and response [21]. Additionally, the tumor microenvironment changes by immune cell infiltration, particularly macrophages with a tumor-promoting phenotype, which interact with adipocytes and cancer cells to support tumor progression [4,20].

**Figure 1:**
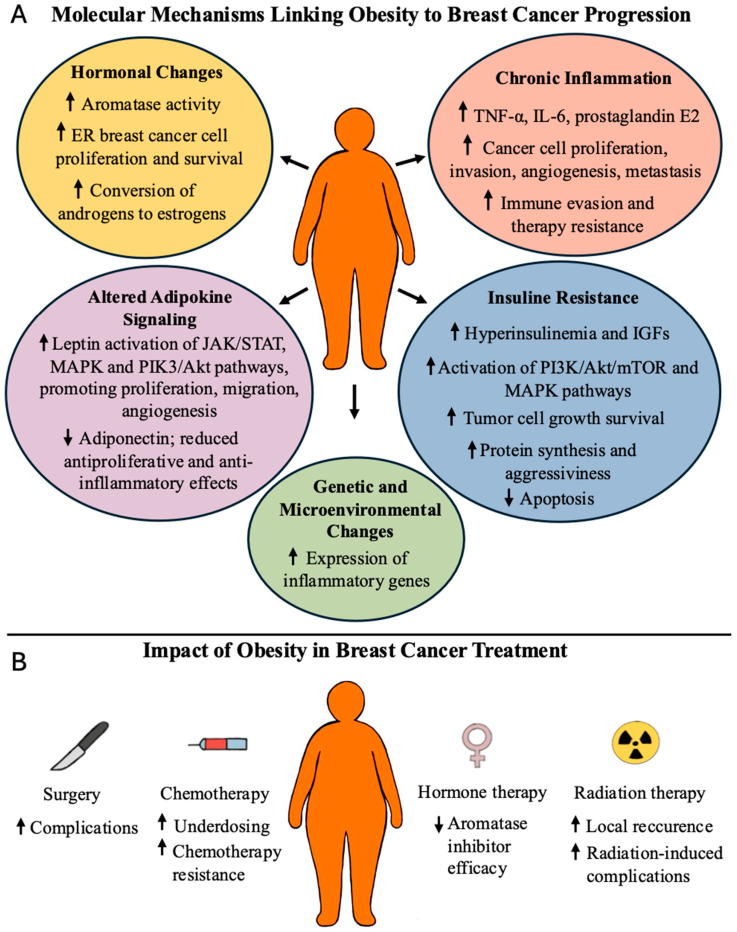
Adverse effects of obesity in BC patients (panel **A**). Adverse effects of obesity in BC cancer treatment (Panel **B**). ↑ denotes increase; ↓ denotes decrease.

In summary, obesity drives BC progression through hormonal imbalance, systemic inflammation, dysregulated metabolism, and stromal–immune interactions, all of which impair treatment efficacy and worsen prognosis (Figure 1).

### 2.4. Influence of Obesity on Breast Cancer Treatment

Obesity negatively affects both the efficacy and safety of BC treatment through multiple mechanisms. Patients with obesity have a higher risk of surgical complications, such as infections, seromas, lymphedema, delayed wound healing, and chronic post-mastectomy pain. They are also less likely to undergo breast reconstruction and experience higher complication rates with implant-based approaches compared to autologous reconstruction [20,21,22].

Among chemotherapy patients with obesity, concerns about increased toxicity often result in dose reductions. However, evidence supports the use of full weight-based dosing to improve survival without increased adverse effects [21]. Adipose-derived leptin may reduce chemosensitivity by inhibiting apoptosis. Similarly, aromatase inhibitors may be less effective in postmenopausal women with obesity due to increased peripheral estrogen production [22]. Obesity also compromises the effectiveness of radiation therapy by increasing local recurrence rates and treatment-related complications [22].

### 2.5. Influence of Weight Loss on Breast Cancer Risk and Prognosis

Weight loss has been shown to reduce the risk of BC in women with obesity without a prior cancer diagnosis. However, evidence of the benefit of weight reduction after BC diagnosis for recurrence or survival remains limited. Table 1 presents major studies on weight loss following diagnosis and its potential impact on recurrence. Although intentional weight loss may offer modest benefit, most interventions using diet and exercise achieve only small reductions in body weight and show weak effects on oncologic outcomes.

In contrast, post-diagnosis weight gain is consistently associated with worse prognosis. A meta-analysis by Playdon et al. involving 23,832 early-stage BC patients showed that weight gain ≥5% was associated with higher all-cause mortality (HR 1.12; 95% CI 1.03–1.22; *p* = 0.01), increasing further at ≥10% gain (HR 1.23; 95% CI 1.09–1.39; *p* < 0.001). It is noteworthy that BC-specific mortality exhibited only a marginal impact (HR 1.17; 95% CI 1.00–1.38; *p* = 0.05) [22]. Unintentional weight loss exceeding 10% within the first year of diagnosis is associated with worse outcomes due to disease progression and muscle wasting [29].

In summary, while weight loss may reduce BC risk in women with obesity, its effect on prognosis after diagnosis remains inconclusive. Nevertheless, both post-diagnosis weight gain and unintentional weight loss are consistently associated with poorer survival outcomes.

### 2.6. GLP-1 Receptor Agonists: Mechanism of Action, Effects, and Side Effects

#### 2.6.1. Mechanism of Action

Understanding GLP-1 RAs requires knowledge of the incretin system. Incretins are gastrointestinal hormones released after food intake that enhance glucose-dependent insulin secretion from pancreatic beta cells, helping regulate postprandial blood glucose levels [30]. The incretin effect describes the amplified insulin response observed with oral versus intravenous glucose due to these hormones [30]. The two primary incretins are GLP-1 and glucose-dependent insulinotropic polypeptide (GIP), both secreted by intestinal cells [30]. GLP-1 exerts additional metabolic benefits by suppressing glucagon secretion, delaying gastric emptying, and reducing appetite. These effects occur through GLP-1 receptors located in the pancreas, gastrointestinal tract, heart, kidneys, and central nervous system.

The central nervous system plays a pivotal role in appetite regulation via GLP-1 receptors. GLP-1 RAs exert anorexigenic effects by modulating neural circuits that regulate satiety and food intake. Upon central or peripheral administration, GLP-1 RAs increase neuronal activity (e.g., c-Fos expression) in the paraventricular nucleus, amygdala, nucleus of the solitary tract, area postrema, and arcuate nucleus, key regions involved in appetite control [31]. Therefore, effective central activity depends on the drug’s ability to cross the blood–brain barrier.

In type 2 diabetes, the incretin effect diminishes because of impaired GLP-1 secretion and decreased GIP activity. Pharmacological agents, specifically GLP-1 RAs and DPP-4 inhibitors, were developed to restore this physiological function. The first newer, longer-acting compounds such as semaglutide, approved in a higher dose (2.4 mg weekly) for obesity treatment in 2021, followed the first-generation agent liraglutide. In fact, semaglutide induces average weight loss of 15–17% and has been associated with cardiovascular protection [32,33].

#### 2.6.2. Cardio Protection of GLP-1 RAs Agonists

GLP-1 RAs, including semaglutide and liraglutide, have shown cardiovascular benefits in patients with type 2 diabetes [34], and emerging evidence suggests tirzepatide—a dual GLP-1/GIP agonist—may offer even greater cardio protection, including among cancer patients [35].

A 2024 retrospective cohort study by Lu et al., using the TriNetX database, evaluated outcomes in BC patients with obesity treated with GLP-1 RAs compared to other weight loss medications. In 380 propensity-matched pairs, those treated with GLP-1 RAs (semaglutide or liraglutide) showed significantly lower risks of all-cause mortality (HR 0.593; 95% CI 0.457–0.77), acute heart failure (HR 0.496), angina (HR 0.566), and stroke (HR 0.661), with no increase in myocardial infarction or arrhythmias [36].

These cardioprotective effects are particularly relevant for BC patients, who often face elevated cardiovascular risk due to cardiotoxic treatments such as anthracyclines or HER2-targeted therapies. Interestingly, GLP-1 RAs have been associated with reduced incidence of congestive heart failure in these patients [37].

#### 2.6.3. New Advances in the Pharmacological Treatment of Obesity in the Context of Bariatric Surgery

Pharmaceutical development has advanced toward dual and triple agonists targeting GLP-1, GIP, glucagon, and amylin pathways to amplify metabolic benefits. Tirzepatide has demonstrated up to 22.5% weight loss in phase 3 trials [38], while other agents like cagrisema (GLP-1/amylin RA) and retatrutide (GLP-1/GIP/glucagon RA) show even greater efficacy in early-phase studies [13].

Comparative studies have confirmed that GLP-1 RAs achieve superior weight loss compared to diet and exercise alone [39], though bariatric surgery remains more effective, albeit with greater surgical risk [40].

#### 2.6.4. Side Effects of GLP-1 RAs

In a recent randomized trial comparing semaglutide and tirzepatide, gastrointestinal symptoms—nausea, vomiting, diarrhea, and constipation—were the most common adverse effects. These were typically mild to moderate and occurred during dose escalation [36]. Gastrointestinal events occurred slightly more often with semaglutide (79.0%) than tirzepatide (76.7%) and were the leading cause of discontinuation: 5.6% versus 2.7% respectively [41].

Other side effects occurring in ≥5% of patients included fatigue, headache, dyspepsia, eructation, sinusitis, and injection-site reactions (more frequent in tirzepatide users). Serious adverse events were rare. One case of pancreatitis occurred in the semaglutide group, with no reported cases of thyroid or pancreatic cancer in either arm [41].

In summary, GLP-1 RAs represent a significant advancement in obesity management, offering substantial weight loss through modulation of physiological appetite and metabolic pathways. When combined with lifestyle interventions, these agents provide a powerful strategy for improving metabolic health in patients with or at risk of BC [39,41].

## 3. Clinical Studies on GLP-1 Agonists in Breast Cancer Patients

Three recent analyses summarized in abstracts have reviewed the use of GLP-1 RAs for weight management in BC patients, with varying results.

Shen et al. [11] conducted a single-center retrospective cohort study involving seventy-five BC patients (median age 52 years) who received a GLP-1 RA between 2015 and 2023. With a median treatment duration of 20 months, patients experienced a mean relative weight loss of 5% at 12 months, corresponding to absolute reductions of 2.8 kg at 6 months, 4.2 kg at 12 months, and 6.2 kg by the end of follow-up. Notably, the weight loss effects were consistent across subgroups stratified by baseline BMI, anti-estrogen therapy, and diabetes status, suggesting broad applicability in routine oncology settings.

Urueta Portillo et al. [10] focused on postmenopausal, hormone receptor-positive patients undergoing endocrine therapy (with either aromatase inhibitors or Selective Estrogen Receptor Modulators [SERMs]). They compared 12-month BMI changes with reference values from non-cancer populations. While semaglutide and liraglutide resulted in mean BMI reductions of 4.3% and 3.5%, respectively, these were markedly lower than the 14% and 8.4% reductions seen in the general population. Dulaglutide and tirzepatide yielded even less benefit (2.3% loss and 2.3% gain, respectively), suggesting that concurrent endocrine therapy may attenuate the weight loss efficacy of GLP-1 RAs in this population.

Fischbach et al. [12] analyzed a large institutional database of 5430 non-metastatic BC patients, seventy of whom received semaglutide or tirzepatide between 2017 and 2022. Treated patients (mean age 58 years, mean BMI 35.9 kg/m^2^) experienced a statistically significant average weight reduction of 3.0 kg (BMI decrease of 1.1 kg/m^2^), with a maximal loss of 8.9 kg (3.2 kg/m^2^) (*p* < 0.0001). Importantly, there were no statistically significant differences in local, locoregional, or distant recurrence rates between treated and untreated patients, offering preliminary reassurance about oncologic safety.

Collectively, these studies support the weight loss efficacy of GLP-1 RAs in BC patients, with reductions ranging from 2.3% to 5% of baseline body weight. Although direct head-to-head comparisons are absent, these findings indicate that weight loss in BC patients is less significant compared to non-cancer populations [41]. One likely explanation is that endocrine therapy creates a metabolic environment characterized by estrogen deficiency, insulin resistance, and inflammation that diminishes the pharmacological effects of GLP-1RAs on weight loss and glucose metabolism in BC patients [20]. In addition, differences in study design—ranging from single-center cohorts to large databases, use of various GLP-1 agents, and diverse comparator strategies—contribute to the variability in outcomes.

## 4. GLP-1 Agonists and Cancer Risk

Currently, limited evidence exists on the direct impact of GLP-1 RA–induced weight loss on BC recurrence or survival. In the study by Fischbach et al., recurrence rates were similar between the treated (1.5% local, 0% locoregional, and 1.5% distant) and untreated groups (1.3%, 4.7%, and 3.3%, respectively), with no statistically significant differences observed [12].

Chen et al. [42] conducted a retrospective cohort study involving 67,591 cancer survivors matched to non-cancer patients. The use of GLP-1 RAs (liraglutide, semaglutide, exenatide, and dulaglutide) was associated with significantly lower all-cause mortality in cancer survivors (HR 0.36; 95% CI 0.25–0.51) and no increase in recurrence risk (HR 0.80; 95% CI 0.50–1.30). These findings affirm the oncologic safety of GLP-1 RAs. However, additional long-term data are required, particularly in terms of the sustainability of weight loss among BC survivors.

Among non-cancer populations, the long-term safety of GLP-1 RAs remains an area of active investigation. Table 2 summarizes the main studies assessing potential cancer-related risks. GLP-1 RA is safe and can lower the risk of certain obesity-related cancers. An exception is a recent study indicating an increased BC risk in non-obese diabetic patients treated with GLP-1 RAs [43]. Overall, however, the evidence does not support an elevated recurrence risk with GLP-1 RA use in BC patients.

GLP-1 receptors are present in thyroid parafollicular C cells, and studies in rodents have demonstrated that high doses of GLP-1 RAs can lead to C-cell hyperplasia and the development of medullary thyroid tumors. Nonetheless, most large-scale human studies do not show a statistically significant increase in thyroid cancer risk with GLP-1 RA use over short- to mid-term follow-up (up to four years) [46]. Although the data are reassuring, it is prudent to exercise caution in individuals with a personal or family history of medullary thyroid carcinoma or MEN2 syndrome.

One important and still unanswered question relates to the potential mechanisms underlying GLP-1RA-related reduction of cancer risk in patients with type 2 diabetes. Song et al. [47], using the TriNetX database, evaluated 726,846 non-cancer patients with type 2 diabetes who received either semaglutide, dulaglutide, or liraglutide. In this study, semaglutide demonstrated the greatest weight loss effect, while exenatide had the least. However, the differences in weight loss did not translate to significant changes in the risk of obesity-related cancers or, for lung cancer, a non-obesity-related tumor. The authors suggested that the reduction in cancer risk may not be mediated by the weight loss but rather by other mechanisms; such potential mechanisms include the anti-inflammatory effects of GLP-1RAs on various pathways [48].

Ongoing pharmacovigilance and post-marketing surveillance remain essential for monitoring rare but serious adverse events.

Table 3 presents practical clinical tips for oncologists managing BC patients with overweight or obesity who are candidates for GLP-1 RA therapy.

## 5. Limitations and Future Directions

While initial findings are promising, there are still several limitations to the use of GLP-1 RAs in BC patients. There is a lack of data defining the optimal timing, dosage, and duration of therapy in this population. The timing of introducing these agents, whether during active treatment or in the survivorship phase, remains undetermined. Additionally, it is unclear if dosing strategies should be different from those employed in the general population.

Furthermore, conflicting evidence from non-cancer populations—particularly regarding BC risk—complicates efforts to use GLP-1 RAs for chemoprevention in high-risk women with overweight/obesity, especially those already receiving hormonal chemoprevention. Extended prospective studies within non-cancer cohorts are essential to elucidate these risks.

To date, safety data on GLP-1 RAs in BC patients remain sparse. There is an urgent need for larger prospective trials to assess efficacy, safety, and long-term oncologic outcomes. Future research should also investigate the biological mechanisms underlying the apparent reduction in GLP-1 RA efficacy among BC patients receiving endocrine therapy. Additional endpoints should include effects on treatment-related toxicity, quality of life, disease-free survival (DFS), and overall survival (OS).

An important barrier to widespread adoption is the prohibitive cost of GLP-1 RAs, which may limit access for underserved populations. A recent study by Hundal et al. [48] highlighted disparities in GLP-1 RA prescribing patterns among BC survivors with diabetes, particularly in non-white women.

In conclusion, GLP-1 RAs represent a novel and potentially valuable tool for weight management in BC patients. Beyond weight loss, these agents offer additional benefits including cardiovascular protection and survival advantages. However, rigorous prospective studies are essential to determine their full role in comprehensive BC management.

## Figures and Tables

**Table 1:** Summary of weight loss and Breast Cancer studies: main studies in the literature that evaluated the effects of weight loss on the risk of Breast Cancer for women without cancer and for prognosis in patients with the diagnosis of Breast Cancer.

Study (First Author, Year)	Intervention	Population
Teras, 2020 [23]	Sustained weight loss over ~10 years	180,885 women ≥ 50 years
Kristensson, 2024 [24]	Bariatric surgery	2867 women, BMI ≥ 38
Chlebowski, 2020 (WHI study) [25]	Low-fat diet, high fruits/vegetables	48,835 postmenopausal women
Goodwin, 2020 (LISA study) [26]	Phone-based lifestyle weight loss program	338 women, HR+ BC on letrozole
Chlebowski, 2006 (WINS study) [27]	Low-fat diet post-BC surgery	2437 women with resected BC
Träsel, 2021 (Meta-analysis) [28]	Specific dietary interventions post-treatment	7 RCTs with 6087 women with early-stage BC patients
Shaikh, 2020 (Cochrane Review) [8]	Weight loss interventions (diet, exercise, and psychosocial)	2028 women, BMI ≥ 25 kg/m^2^

Abbreviations: BMI: body mass index; HR+: hormone receptor positive; RCT: Randomized Controlled Trial; BC: Breast Cancer.

**Table 2:** Summary of GLP-1RA studies and overall BC risks.

Study (First Author, Year)	Study Design	Population	Exposure	Clinical Outcome	Implication
Funch, 2018 [44]	Real-world observational study	188,550 women using liraglutide	Liraglutide	No increased risk of BC with liraglutide use (HR 0.90; 95% CI: 0.67–1.22)	Supports safety of liraglutide regarding BC risk
Cheng, 2025 [43]	Retrospective cohort using Epic Cosmos database	4014 adults with type 2 diabetes (no prior BC), 2010–2020	GLP-1 RAs vs. various antidiabetics	Higher BC incidence with GLP-1 RAs (HR 1.19; 95% CI (1.14–1.24)	Possible link between GLP-1 RAs and BC incidence; further study needed
Lucas Mavromatis, 2025 [45]	Propensity-matched cohort	85,015 adults with obesity and T2DM	GLP-1 RAs vs. DPP-4 inhibitors	Lower risk of obesity-related cancers (HR 0.93; 95% CI 0.88–0.98) and mortality (HR 0.92; 95% CI 0.87–0.97); stronger mortality benefit in women (HR 0.80; 95% CI, 0.74–0.86)	GLP-1 RAs may reduce cancer risk and improve survival in diabetic patients with obesity

Abbreviations: BC: Breast Cancer; 95% CI: 95% confidence interval; HR: Hazard Ratio; T2DM: Type 2 Diabetes Mellitus; DDP-4: dipeptidyl peptidase 4; GLP-1 RAs: glucagon-like peptide-1 receptor agonists.

**Table 3:** Tips for medical oncologists on the Use of GLP-1 Agonists in BC Patients.

Clinical Issue	Practical Tip
Patient Selection	Consider GLP-1RAs (e.g., semaglutide and tirzepatide) in BC patients with obesity, especially with comorbidities such as diabetes, insulin resistance, or NAFLD.
Timing of Use	GLP-1RA initiation can occur during survivorship care or adjuvant endocrine therapy, especially in cases of treatment-induced weight gain.
Multidisciplinary Care	Collaborate with endocrinologists or obesity specialists when introducing GLP-1RAs.
Monitoring and Safety	Watch for GI side effects, thyroid risks, and pancreatitis. Monitor bone health and metabolic effects, especially with endocrine therapies.
Drug Interactions	Review all medications for interactions. Space oral meds at least 1 h before GLP-1RA injection. Monitor for altered efficacy of tamoxifen, CDK4/6 inhibitors, and bisphosphonates.
Glycemic Control	In diabetic/pre-diabetic patients, adjust antidiabetic regimens to avoid hypoglycemia. Monitor glucose and HbA1c.
Oncologic Vigilance	Track tumor markers, imaging, and unexpected changes in drug efficacy. Avoid in patients with MEN2 or medullary thyroid carcinoma history.
Long-term Monitoring	Track thyroid function, GI symptoms, and document long-term outcomes, particularly if therapy exceeds 12 months.
Lifestyle Integration	Use GLP-1RAs alongside, not in place of diet, exercise, and behavioral interventions.
Patient Education	Discuss treatment benefits, risks, administration, duration, cost, and expected weight outcomes to ensure shared decision making.
Follow-Up	Reassess weight loss goals, glycemic status, and quality of life every 3–6 months. Adjust treatment if no meaningful benefit.
Documentation	Record BMI, baseline labs, treatment timelines, adverse events, and cancer-specific outcomes for clinical and research use.

Abbreviations: NAFLD: Nonalcoholic Fatty Liver Disease; MEN2: Multiple Endocrine Neoplasia Syndrome type 2; GLP-1 RAs: glucagon-like peptide-1 receptor agonists; BMI: body mass index.

## Data Availability

No new data were created or analyzed in this study. Data sharing is not applicable to this article.

## Abbreviations

The following abbreviations are used in this manuscript:

95% CI	95% Confidence Interval
BC	Breast Cancer
BMI	Body Mass Index
CDK4/6	Cyclin-Dependent Kinase 4/6 Inhibitors
c-Fos	Proto-oncogene c-Fos
DFS	Disease-Free Survival
DPP-4	Dipeptidyl Peptidase 4
GI	Gastrointestinal
GIP	Glucose-Dependent Insulinotropic Polypeptide
GLP-1	Glucagon-Like Peptide-1
GLP-1 RAs	GLP-1 Receptor Agonists
HbA1c	Hemoglobin A1c
HR	Hazard Ratio
IGFs	Insulin-Like Growth Factors
IL-6	Interleukin 6
JAK/STAT	Janus Kinase/Signal Transducer and Activator of Transcription
MAPK	Mitogen-Activated Protein Kinase
MEN2	Multiple Endocrine Neoplasia Syndrome type 2
mTOR	Mammalian Target of Rapamycin
NAFLD	Nonalcoholic Fatty Liver Disease
OS	Overall Survival
PI3K/Akt	Phosphatidylinositol 3-Kinase/Protein Kinase B
RCTs	Randomized Controlled Trials
SERMs	Selective Estrogen Receptor Modulators
T2DM	Type 2 Diabetes Mellitus
TNF-α	Tumor Necrosis Factor Alpha
